# Supplementary material for: Transcriptome Analysis Reveals Vimentin-Induced Disruption of Cell–Cell Associations Augments Breast Cancer Cell Migration
Source: Cells. 2022 Dec 13;11(24):4035. doi: 10.3390/cells11244035 (PMC9776984; doi:10.3390/cells11244035)
Supplement: Supplementary file 1 [file cells-11-04035-s001.zip › DEGs and enrichment analysis.zip!/DEGs and enrichment analysis/WT_VIMvsVector KEGG/ALL/WT_VIMvsVector_ALL.pathway_map.zip!/WT_VIMvsVector_ALL.pathway_map/src/hsa04066.html]

hsa04066


- hsa:6513

- Up regulated genes

ENSG00000117394(1.94254957435037)

- hsa:6513

- Up regulated genes

ENSG00000117394(1.94254957435037)

- hsa:2023

- Down regulated genes

ENSG00000186197(-1.71414885497902)

- hsa:1906

- Down regulated genes

ENSG00000078401(-3.03994258238563)

- hsa:596

- Up regulated genes

ENSG00000171791(2.03324203294485)

Close
